# Cefepime-Induced Generalized Fixed Drug Eruption With Morbilliform Rash

**DOI:** 10.7759/cureus.53011

**Published:** 2024-01-26

**Authors:** Grayson P Clark, Haley M Caldwell, Christopher A Coop, Brittanie I Neaves, Peter W Barnes

**Affiliations:** 1 Medicine, F. Edward Hebert School of Medicine, Uniformed Services University of the Health Sciences, Bethesda, USA; 2 Internal Medicine, Keesler Medical Center, Biloxi, USA; 3 Allergy and Immunology, Keesler Medical Center, Biloxi, USA; 4 Dermatology, Keesler Medical Center, Biloxi, USA

**Keywords:** cd8+ t cell activation, cefepime, morbilliform rash, violaceous patches, fixed drug eruptions

## Abstract

Fixed drug eruption (FDE) is a cutaneous reaction that characteristically recurs in the same locations upon re-exposure to the offending drug(s). The typical presentation of FDEs is single or multiple violaceous plaques with hyperpigmentation due to inflammation. The causative agents for FDEs include antibiotics, nonsteroidal anti-inflammatory drugs (NSAIDs), acetaminophen, barbiturates, and anticonvulsants. We present an interesting case of a generalized fixed drug eruption secondary to cefepime that resolved with the cessation of the offending drug and the institution of antihistamines and topical steroids.

## Introduction

Fixed drug eruptions (FDEs) manifest with characteristic erythematous to violaceous plaques, which may become bullous in the center. The lesions occur at the same location upon re-exposure to the culprit drug. When the lesions heal, a residual hyperpigmentation remains [1]. The pathophysiology of FDEs is mediated through the activation of effector-memory intraepidermal cluster of differentiation (CD) 8+ T cells. These T cells express cytotoxic granules that result in the dermatological findings seen in FDEs [1]. The lesions on examination come from the further recruitment of CD4+ and CD8+ T cells. These CD8+ T cells remain dormant in healed lesions until the re-administration of the offending drug; then, the CD8+ T cells reactivate and release interferon gamma, perforin, and granzyme B [1-3]. Previous studies have established the relationship between FDEs and other beta-lactam antibiotics. We report on a unique generalized fixed drug eruption secondary to cefepime.

## Case presentation

A 63-year-old male retired US military service member presented to the emergency department with pain and drainage in his left ankle for one week after an open reduction internal fixation (ORIF). He described the pain as 8/10 and sharp, localized to the medial and lateral aspects of his left ankle with serous drainage. He also reported nausea, chills, and headache without fever, vomiting, chest pain, or shortness of breath.

His past medical history was pertinent for type 2 diabetes mellitus, hypertension, and hyperlipidemia, all well controlled with metformin 500 mg daily, lisinopril 20 mg daily, and atorvastatin 40 mg daily, respectively. He had a methicillin-susceptible *Staphylococcus aureus* infection in his left elbow in 2016, treated with cefazolin 8 g IV for six weeks. He had documented allergic reactions that include flushing, urticaria, and maculopapular rashes to cephalexin, clindamycin, morphine, sulfamethoxazole-trimethoprim (TMP-SMX), penicillin, meperidine, and oxycodone. In the emergency department, he was given ketorolac 15 mg IV, vancomycin 2 g IV over two hours, and piperacillin-tazobactam 4.5 g IV and was admitted to internal medicine for cellulitis.

**Figure 1:**
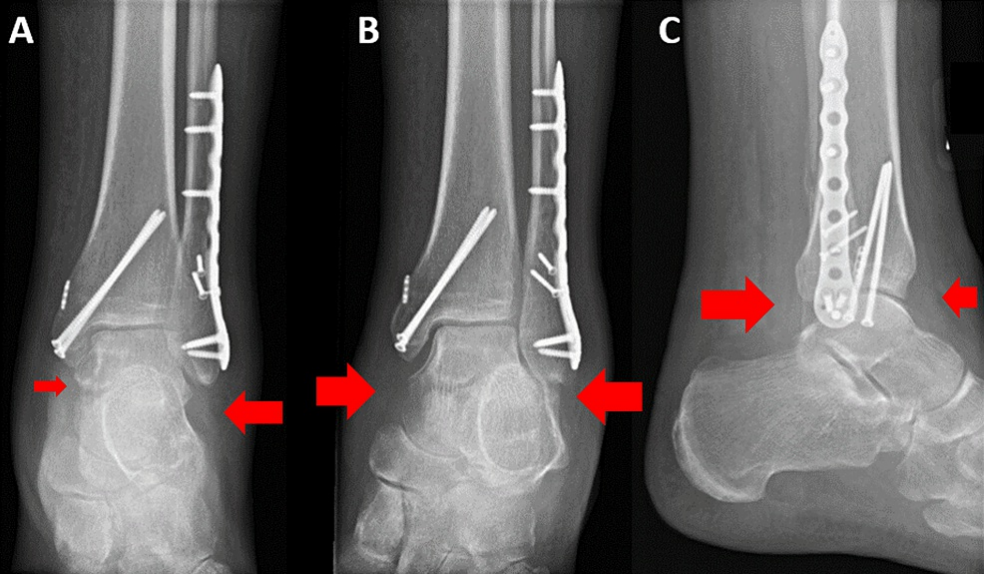
Radiographs taken in the emergency department of the patient's left ankle with anteroposterior (A), mortise (B), and lateral views (C) with red arrows indicating soft tissue swelling.

His vitals were within normal limits without signs of sepsis. His laboratory results were significant for an erythrocyte sedimentation rate of 57 mm/hour and C-reactive protein of 1.00 mg/dL without leukocytosis. An X-ray of his left ankle revealed soft tissue swelling with evidence of his recent ORIF (Figure 1).

**Figure 2:**
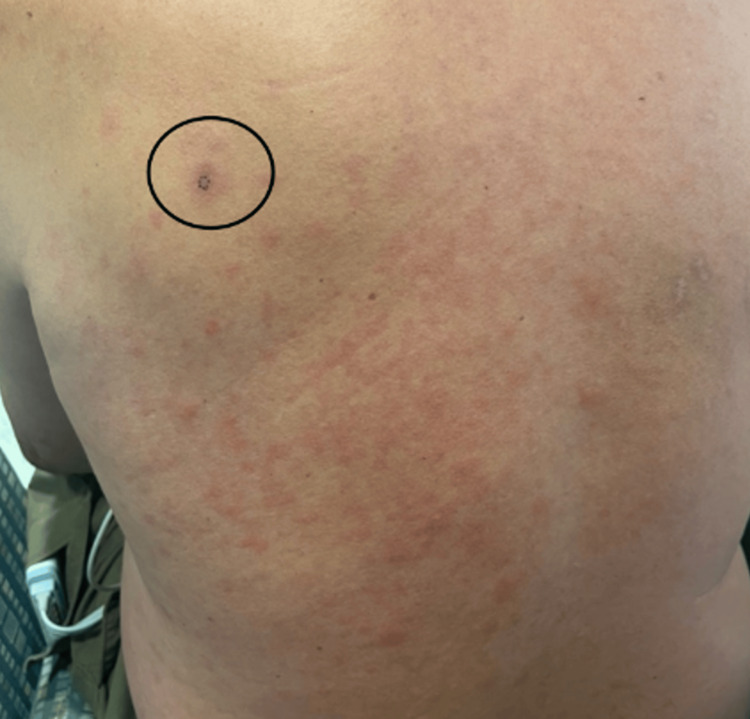
Morbilliform rash on the patient's back that erupted after the administration of cefepime. Circled is the violaceous patch from which a punch biopsy was taken.

Upon admission, the vancomycin was continued, and the piperacillin-tazobactam was switched to cefepime 2 g IV. There was concern over prior reactions to penicillin, and the infectious disease service recommended cefepime. Over the next few hours, he began to develop mild periorbital edema and a pruritic, erythematous, morbilliform rash on his neck, back, and upper chest (Figure 2). He confirmed that he has allergies to several medications, but this presentation was unlike any of his previous medication reactions.

The next morning, it was noted that his periorbital edema was increasing, and his rash was improving but remained erythematous. He started cetirizine 10 mg twice daily and diphenhydramine 25 mg every six hours as needed. His vancomycin was adjusted to deliver 1000 mg over 90 minutes as it was suspected that the rash was due to antibiotics. He had begun to tolerate weight-bearing on his left foot and was receiving acetaminophen 650 mg every four hours.

**Figure 3:**
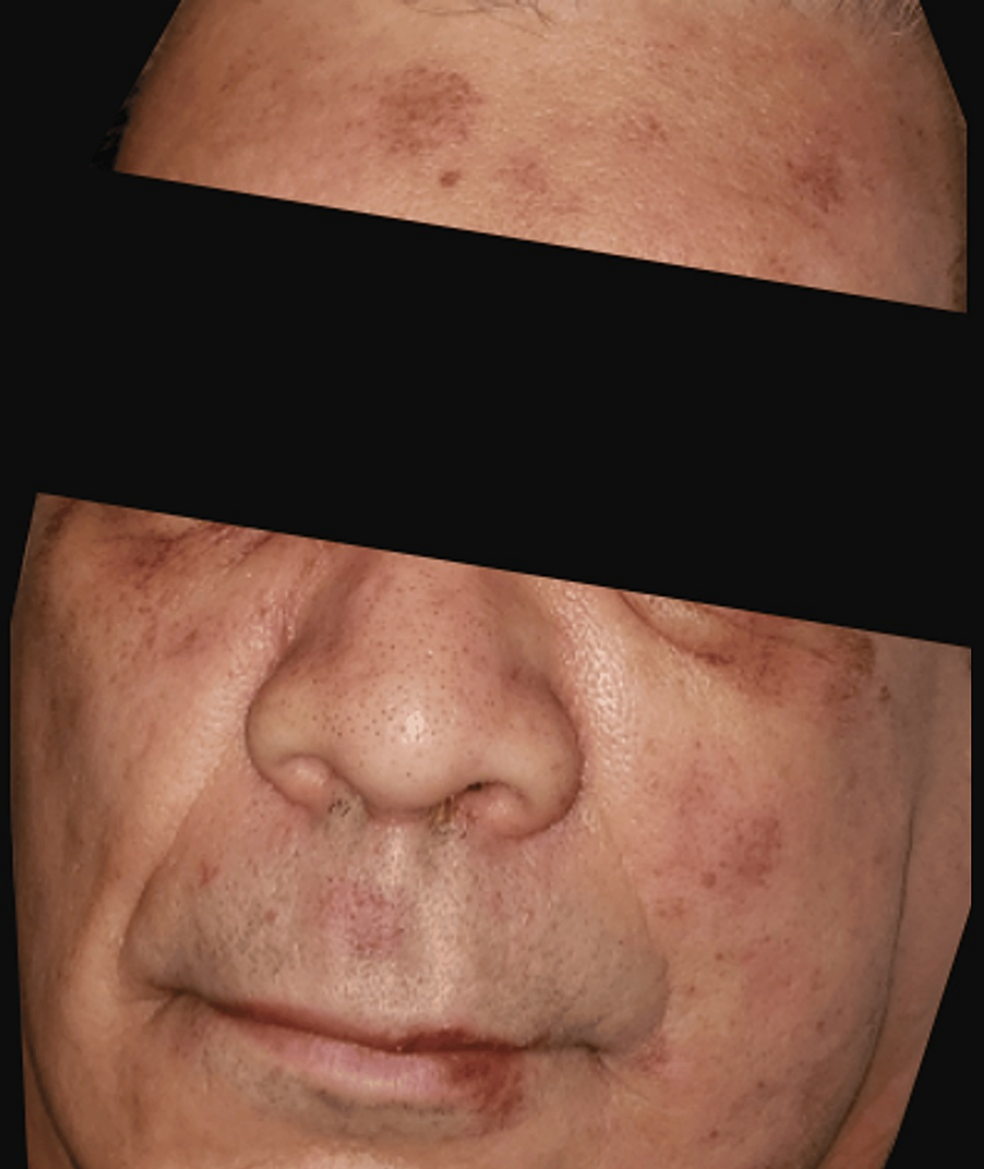
Violaceous patches on the patient's forehead, bilateral lower eyelids, left cheek, and lip.

**Figure 4:**
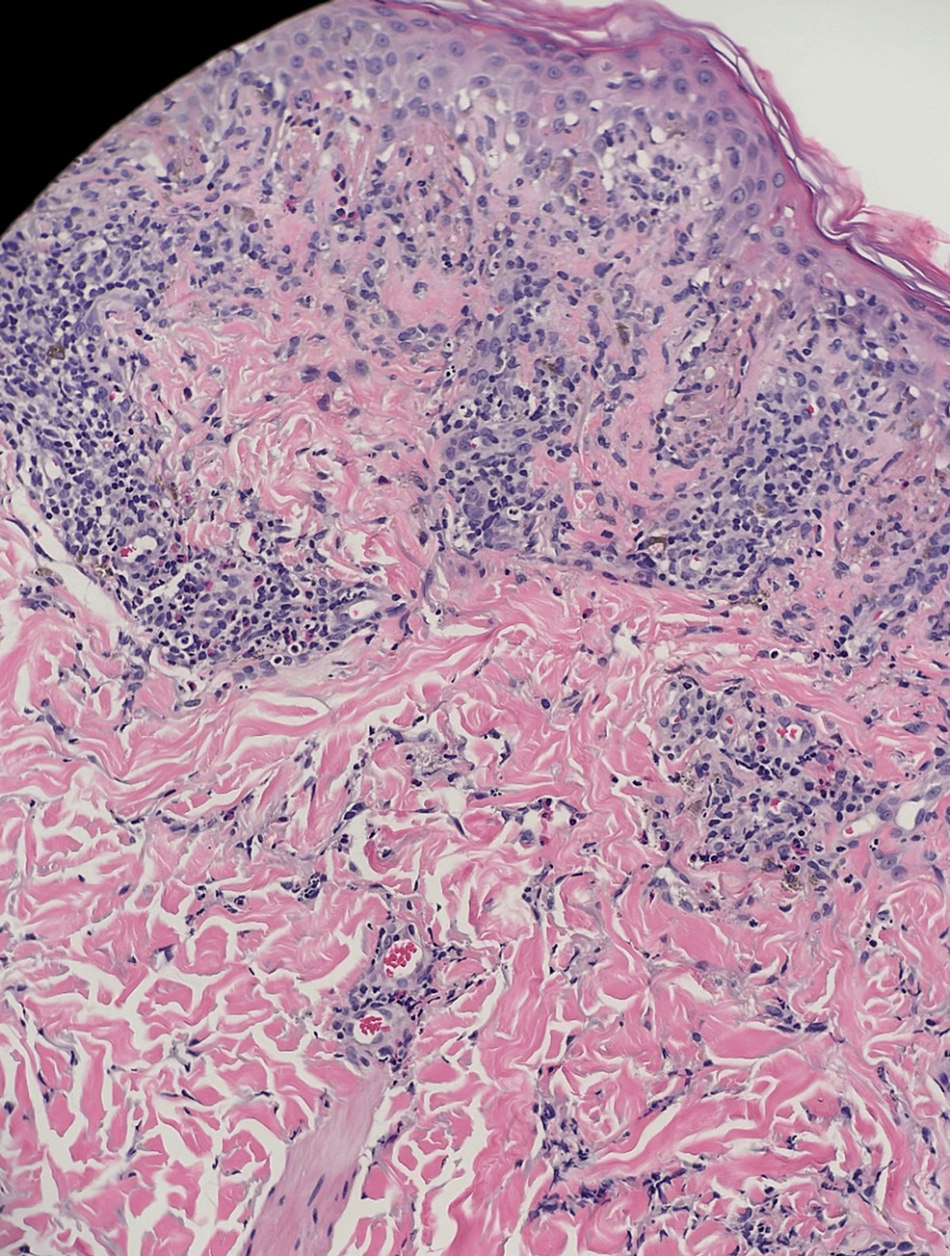
Skin biopsy from the patient's left upper back showed dyskeratotic cells within the epidermis with eosinophils in the epidermis and superficial dermis, along with superficial dermal perivascular lymphohistiocytic inflammatory infiltrate.

His rash continued the next day, and he developed annular violaceous patches with an erythematous rim on his cheeks and forehead, as well as a solitary patch on his left upper back (Figures 3, 4). Dermatology was consulted for possible drug eruption and a punch biopsy was taken from the violaceous patch on his left upper back. His skin biopsy showed dyskeratotic cells within the epidermis with eosinophils in the epidermis and superficial dermis (Figure 4). Also present was superficial dermal perivascular lymphohistiocytic inflammatory infiltrate with occasional neutrophils. This was consistent with generalized fixed drug eruption (FDE) given these findings coupled with his history. The suspected offending agent was cefepime.

Cefepime and vancomycin were discontinued, and the patient was switched to delafloxacin 450 mg every 12 hours. He was able to fully tolerate weight-bearing and ambulation without assistance. His erythema and induration on his left ankle improved, and the rash also began to resolve. He was discharged the following morning with five days of delafloxacin for cellulitis, topical desonide 0.05% cream for his face, and topical triamcinolone 0.1% cream for his trunk. The patient was seen two weeks later for a follow-up with the resolution of his FDE. An allergy consult was accomplished, and penicillin skin testing was not recommended for an FDE. Patch testing was considered, but it was not done because of the lack of sensitivity of patch testing with FDEs.

## Discussion

FDEs are cutaneous reactions that occur in the setting of an offending drug. The characteristic presentation of FDE is multiple or single violaceous plaques with hyperpigmentation due to inflammation after resolution. These lesions typically occur at the same anatomical site that was exposed to the agent. Understanding the pathophysiology and management of FDEs is imperative for clinicians, as FDEs can greatly impact patient care.

The most common type of drug reaction is the morbilliform exanthem, accounting for up to 95% of all skin reactions with an identifiable offending drug [4]. FDEs are much less common than the morbilliform exanthem reactions, with a calculated incidence of 0.003% [5]. The studied causative agents for FDEs include antibiotics (TMP-SMX, tetracyclines, antifungals, penicillins, quinolones, and dapsone), nonsteroidal anti-inflammatory drugs (NSAIDs), acetaminophen, barbiturates, and anticonvulsants [6,7]. There are case reports of FDEs secondary to third-generation cephalosporins but none to fourth-generation cephalosporins [8,9]. We report on a unique generalized fixed drug eruption secondary to cefepime with a morbilliform rash.

Although the pathophysiology of FDEs is not completely understood, the working theory depicts mediation through CD8+ T cells. It is hypothesized that the activation of resident effector-memory intraepidermal CD8+ T cells in response to the drug triggers the lesion through the expression of cytotoxic granules [1]. The activation of these CD8+ T cells is influenced by mast cells through tumor necrosis factor-alpha, which modulates cell adhesion molecules on adjacent keratinocytes [2].

The violaceous lesions observed on physical examination manifest from the further recruitment of CD4+ and CD8+ T cells, which damages tissue [1]. These CD8+ T cells remain dormant in healed lesions until the re-administration of the offending drug. When re-administered, the CD8+ T cells reactivate again to release interferon gamma, perforin, granzyme B, and other cytotoxic granules [2,3,10]. Hemostasis and immune modulation are achieved with the recruitment of regulatory T cells (Treg). Most of the activated CD8+ T cells causing the damage undergo apoptosis to facilitate their removal, but memory T cells remain in the location as a minority of intraepidermal CD8+ T cells are protected from apoptosis by interleukin (IL) 15 from basal keratinocytes. In the late stage of the immune response, CD4/CD25/forkhead box P3+ (Foxp3+) regulatory T cells (Treg) are recruited into the lesions and take part in the homeostatic control of the immune reaction [1-3]. In generalized FDEs, the typical presentation is multiple, disseminated lesions on the trunk and extremities that tend to spare mucosa. In this patient, the violaceous plaque on his trunk was preceded by a morbilliform rash with urticaria, which is atypical of generalized FDEs. Other diagnoses that may present like an FDE include early Stevens-Johnson syndrome, cellulitis, or herpes zoster.

Patch testing can be used to confirm the diagnosis of an FDE. The suspected drug can be applied to an old FDE lesion to elicit a local reaction. Patch testing is safe because the reaction is localized. However, there is no standardized method for patch testing in FDE, and the sensitivity is poor [11,12].

The cornerstone in FDE management is the avoidance of the underlying drug that caused the reaction. Once the drug is withdrawn, the lesions typically resolve within days, but the characteristic hyperpigmentation can remain for weeks to months. From this point, the patient should be advised to avoid the drug or other drugs that share a chemical resemblance. The treatment of FDEs also focuses on the symptomatic relief of pruritus. Topical corticosteroids and antihistamines can be used for patients with single or few lesions, while systemic corticosteroids can be beneficial for those with generalized FDE, although more investigation should be dedicated to this topic.

## Conclusions

In conclusion, this is a unique case report of an FDE from cefepime. The prompt recognition and removal of the culprit drugs are of great importance in the treatment of FDEs. The activation of resident effector-memory intraepidermal CD8+ T cells is the purported mechanism of FDEs. Skin biopsy can be helpful for the diagnosis of FDEs. Fortunately, our patient's course proceeded fortuitously with the cessation of the offending drug and the institution of antihistamines and topical steroids.
